# A study of 82 extended HLA haplotypes in *HFE*-C282Y homozygous hemochromatosis subjects: relationship to the genetic control of CD8+ T-lymphocyte numbers and severity of iron overload

**DOI:** 10.1186/1471-2350-7-16

**Published:** 2006-03-01

**Authors:** Eugénia Cruz, Jorge Vieira, Susana Almeida, Rosa Lacerda, Andrea Gartner, Carla S Cardoso, Helena Alves, Graça Porto

**Affiliations:** 1 Clinical Hematology, Santo António General Hospital, Porto, Portugal; 2 Molecular Immunology and Pathology, Abel Salazar Institute for the Biomedical Science (ICBAS), Porto, Portugal; 3 Iron Genes and the Immune System (IRIS), Institute for Molecular and Cell Biology (IBMC), Porto, Portugal; 4 Molecular Evolution, Institute for Molecular and Cell Biology (IBMC), Porto, Portugal; 5 Molecular Genetics, North Histocompatibility Center, Porto, Portugal

## Abstract

**Background:**

It has been recently demonstrated that CD8+ T-lymphocyte numbers are genetically transmitted in association with the MHC class I region. The present study was designed with the objective of narrowing the region associated with the setting of CD8+ T-lymphocyte numbers in a population of C282Y homozygous hemochromatosis subjects, in whom a high prevalence of abnormally low CD8+ T-lymphocyte counts has been described.

**Methods:**

The study includes 43 C282Y homozygous subjects fully characterized both phenotypically and genotypically. Clinical characterization includes measurements of iron parameters at diagnosis (transferrin saturation and serum ferritin), total body iron stores and T-cell immunophenotyping determined by flow cytometry. Genetic characterization includes HLA class I alleles (A, B and C) and four additional microsatellite markers (D6S265, D6S2222, D6S105 and D6S2239) spanning 5 Megabases in the 6p21.3 region.

**Results:**

Eighty-two extended C282Y carrying haplotypes were defined. Single-locus analysis revealed that the HLA-A region was associated with CD8+ T-cell numbers. Multivariate analysis showed that the combinations of the most common HLA-A alleles (HLA-A*03, -A*02 and -A*01) were associated with significantly lower numbers of CD8+ T-lymphocytes (0.30 ± 0.14 × 10^6^/ml), in comparison with subjects carrying only one copy of those alleles (0.46 ± 0.19 × 10^6^/ml) and subjects without any copy of those alleles (0.79 ± 0.15 × 10^6^/ml;*p *= 0.0001). No differences were observed in CD8+ T-cell counts among control subjects carrying the same combinations of HLA-A alleles (0.47 ± 0.14; 0.45 ± 0.21 and 0.41 ± 0.17 × 10^6^/ml, respectively), therefore not supporting a direct effect of HLA specificity but rather an indirect association with a locus close to HLA-A. Multivariate analysis showed that the combination of the most common HLA-A alleles also have an impact on the clinical expression of HH in terms of iron stores, in males(*p *= 0.0009).

**Conclusion:**

The present study provides evidence supporting an inextricable link between extended HLA haplotypes, CD8+ T-lymphocyte numbers and severity of iron overload in hereditary hemochromatosis(HH). It gives additional information to better define a candidate region involved in the regulation of CD8+ T-lymphocyte numbers. A new evolutionary hypothesis concerning the inheritance of the phenotype of low CD8+ T-lymphocyte numbers associated with particular ancestral HLA haplotypes carrying the C282Y mutation and its implication on the clinical heterogeneity of HH is discussed.

## Background

There are multiple regulatory mechanisms influencing peripheral blood lymphocyte numbers that are responsible for the maintenance of cell numbers in homeostasis [[1] and reviewed in ref [2]]. An increasing number of studies in the last years, both in humans and animal models, point to the involvement of genetic factors in the regulation of CD4 and CD8 T-lymphocyte numbers. In mice, the first study addressing this question showed that the phenotype of low CD4/CD8 ratio was a dominant Mendelian character and was significantly influenced by age [3]. Later studies in mice showed a locus regulating CD4/CD8 ratios localized in or near the TCR α-chain complex region and a significant association with the Major Histocompatibility Complex (MHC) region [4]. Moreover, differences in CD4/CD8 ratios could be explained by variations in the process of CD4 versus CD8 lineage commitment in the thymus [5]. While in mice CD4/CD8 ratios appear to be independent of thymic intrinsic factors [5], in rats CD4/CD8 ratios are determined by thymic factors intrinsic to the strain [6], supporting the hypothesis that such factors are genetically determined.

Studies in humans have also revealed a genetic regulation for CD4/CD8 numbers. Complex segregation analysis of the CD4/CD8 ratios in nuclear families, suggested a major recessive locus with a polygenic component and additional environmental effects for the control of CD4/CD8 ratios [7]. The two components of this ratio were later shown to be regulated by major recessive genes controlling CD4 and CD8 absolute counts, with a residual multifactorial component [8]. A study in monozygotic and dizygotic twins indicates that T-lymphocyte subpopulations have a main genetic control with unique environmental component and significantly influenced by age [9]. More recent efforts to find genes associated with the regulation of lymphocyte numbers and subpopulations have been made using whole genome screen approaches [10,11]. Using 15 *Center D'Etude Du Polymorphisme Humain *(CEPH) families it was shown that lymphocyte counts, B cells, natural killer cells, and CD4 and CD8 counts, were all under significant genetic control, with the CD8+ T-cell counts being the most heritable trait [10]. Significant levels of linkage were found between these cell counts and chromosomes 1, 2, 3, 4, 8, 9, 11, 12 and 18. No linkage was found for chromosome 6, where the MHC is located, although the authors discuss that the HLA region is still an obvious candidate region [10]. Using 405 pairs of dizygotic twins at ages of 12, 14 and 16, it was found suggestive evidence of linkage on chromosome 11p at ages of 12 and 14 [11]. In addition, suggestive evidence of linkage was found in other areas of the genome including chromosome 6q (microsatellite markers D6S1031-D6S462) [11]. All these studies constitute good evidence showing a genetic regulation for CD4 and CD8 numbers although do not provide evidence to confirm the linkage to MHC found in animal models. The involvement of the MHC region on this regulation was first demonstrated in humans in our recent study of the genetic transmission of CD8+ T lymphocytes [12]. The approaches used in that study included association studies between the CD4+ and CD8+ T-lymphocyte counts and the HLA class I alleles (A and B) in an unrelated healthy population, and a sibpair analysis using a population of HLA class I and *HFE *genotyped subjects belonging to hereditary hemochromatosis (HH) families [13]. A strong significant association of the HLA-A*01 with high numbers of CD8+ T cells was found in the unrelated healthy population [12]. Sibpair correlation in families showed a strong impact of the HLA-*HFE *haplotypes on the setting of CD8+ T-lymphocyte numbers but not of CD4+ [12]. Based on these results and also on our previous results showing an association between low CD8+ T-lymphocyte numbers and a more severe expression of iron overload in HH [14-17], we further advanced the hypothesis that gene(s) contributing to the clinical heterogeneity observed in HH could be genes that also control CD8+ T-cell numbers [12]. This might partially explain the great clinical heterogeneity observed in HH patients [18-22] and further explain the possibility that modifier genes, other than *HFE*, are responsible for this phenotypic variation, in accordance with other studies [23,24].

In the present study we attempt to narrow the region associated with the setting of CD8+ T-lymphocyte numbers using a population of C282Y homozygous subjects fully characterized both phenotypically and genotypically. Multivariate models were used to test the association between several genetic markers at the MHC region and CD8+ T-lymphocyte numbers, as well as the impact of these genetic markers on the severity of iron overload in HH. We discuss the hypothesis that the phenotype of low CD8+ T-lymphocyte numbers in HH is inherited in association with particular ancestral HLA haplotypes carrying the C282Y mutation.

## Methods

### Study population

#### C282Y homozygous subjects

A total of 43 hereditary hemochromatosis probands, all homozygous for the C282Y mutation, identified between 1985 and 2004 and regularly followed up at the Hemochromatosis Outpatient Clinic of Santo António General Hospital (HGSA), Porto and at Predictive and Preventive Genetic Center, Porto, have been included in this study. The subjects are all Caucasian from the North of Portugal and include 32 males (mean age at diagnosis = 50 ± 14 years; range 23–75) and 11 females (mean age at diagnosis = 47 ± 12 years; range 32–65) (Table 1). In 25 probands, C282Y homozygozity was detected in the context of suggestive clinical picture of hemochromatosis, generally with related clinical manifestations and in 18, C282Y homozygozity was detected accidentally after a routine test or in the context of a population screening for HH, in whom transferrin saturation (TfSat) and/or serum ferritin were abnormally elevated according to local reference values [25,26] and generally asymptomatic. Clinical, laboratory and genetic data included in this work were partially reviewed from the clinical files of the subjects or were determined for this study, with the informed consent of the subjects according to the Helsinki declaration. The study design was approved by the Hospital Ethical Committee. Clinical and laboratory data from patients have been published elsewhere [16,27,28].

**Table 1:** General characterization of C282Y homozygous probands at the time of diagnosis according to gender

	**Males n = 32**	**Females n = 11**	p*
**Age (years)**	51 ± 13 (29–75)	47 ± 11 (32–65)	n.s.
**Transferrin Saturation (%)**	92 (87–97)	83 (75–92)	n.s.
**Ferritin (ng/ml)**	2694 (2006–3383)	1353 (122–2584)	n.s.
**Total Body Iron Stores (g)**	9.86 (8.19–11.54)	6.11 (3.21–9.02)	0.030

**Total lymphocytes (×10^6^/ml)**	1.91 (1.69–2.13)	2.30 (1.92–2.67)	n.s.
**Total CD4+ cells(×10^6^/ml)**	0.98 (0.84–1.11)	1.09 (0.85–1.32)	n.s.
**Total CD8+ cells(×10^6^/ml)**	0.35 (0.28–0.42)	0.48 (0.36–0.59)	n.s.

Parameters are expressed as mean (95% confidence intervals) except age, which is expressed as mean ± standard deviation (maximum – minimum values).

Estimation of TBIS was obtained in 27 males and 9 females.

*Statistically significant differences between groups (p) were tested using Student's T test.

Severity of HH was evaluated by the calculation of exchangeable iron stores. Thirty-six patients had completed a weekly phlebotomy program until complete iron depletion was achieved (TfSat below 10% and/or ferritin levels below 10 ng/ml). Total body iron stores (TBIS) were *a posteriori *estimated, based on the total amount of hemoglobin removed by phlebotomies with correction for the amount of iron absorbed during the treatment period, as described [29]. In 7 probands (5 males and 2 females) TBIS were not included because patients were treated in other centers and it was not possible to accurately estimate TBIS.

#### Control population

Data from 264 apparently healthy unrelated subjects, previously analyzed by Cruz and co-workers [12], were included in this study. The population consisted of 116 males (mean age 43 ± 13 years, range 20 to 76) and 148 females (mean age 45 ± 13 years, range 18 – 88), from whom the previously determined total CD4+ and CD8+ T-cell numbers, *HFE *genotype and HLA-A, -B and -C genotype were available. This population was used to compare CD8+ T-lymphocyte numbers according to the HLA-A and -B class I alleles between controls and C282Y homozygous subjects. In addition, control subjects carrying the HLA-A*01 (n = 47) were genotyped for the microsatellite markers D6S2222 and D6S105. This subpopulation was used to compare the allele frequencies of these microsatellites between controls and C282Y homozygous, all carrying the HLA-A*01.

### Genetic characterization of C282Y homozygous subjects and haplotype definition

**Figure 1:**
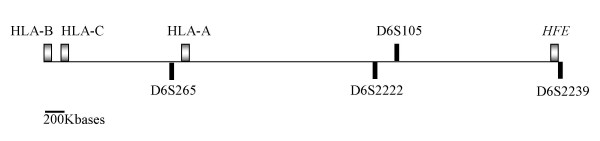
Physical map of the polymorphic markers used in the present study and their relative localization (at scale) in relation to HLA-A, B and C and *HFE*.

All probands were previously genotyped for the *HFE *mutations (H63D and C282Y) and were all homozygous for the C282Y mutation. HLA class I alleles (A, B and C) were available in all probands except the HLA-C in 3 subjects. In addition, four polymorphic markers in the MHC class I region were selected for the definition of extended HLA haplotypes. These include microsatellites spanning 5 Megabases in the 6p21.3 region, from the HLA-B to the *HFE*: D6S265, D6S2222, D6S105, and D6S2239 (Figure 1).

HLA A-B haplotypes and extended HLA haplotypes (including the 4 microsatellite markers) were defined in C282Y homozygous subjects by segregation in families. Haplotypes were defined from the combination of alleles inherited in blocks from each parent or from informative siblings, when any of the parents was not available. Genetic markers included in extended HLA haplotypes were aligned according to physical map of the region as follows: HLA-B, HLA-C, D6S265, HLA-A, D6S2222, D6S105, C282Y mutation, D6S2239 (Figure 1). A total of 86 HLA A-B haplotypes and 82 extended HLA haplotypes could be defined. In two probands the microsatellite genotyping could not be performed because there was no DNA available. Both probands had the HLA A-B haplotypes: A*03-B*07 and A*02-B*51.

### Laboratory methods

Biochemical parameters of iron status used in this work include determinations, at the time of the diagnosis, of TfSat and serum ferritin. These parameters are routinely determined by standard techniques at the Clinical Chemistry Laboratory of HGSA, Porto, as previously described [28]. The T-cell immunophenotype analyzed include total lymphocyte counts and total CD4+ and CD8+ T-lymphocyte counts. Total lymphocyte counts were determined in an automatic blood cell counter (Coulter Gen S) and the CD4 and CD8 + T-lymphocyte counts were determined by flow cytometry, as described in detail elsewhere [12]. *HFE *genotyping was performed using a commercial kit, the Haemochromatosis StripASSAY^B ^(Vienna Lab, Vienna, Austria) as described before [12]. HLA-A and B alleles were detected using low-resolution DNA-based techniques (PCR/sequence-specific oligonucleotide probes, Dynal RELI™ SSO) as previously described [12]. Microsatellite genotyping was performed at the North Histocompatibility Center, Portugal. Genomic DNA was used and the genotyping was done in an ABIPrism 3100 Avant DNA Sequencer. Fluorescent labeled primers were obtained from ABI and were: for D6S265 5'-acgttcgtacccattaacct-3' (forward primer) and 5'-atcgaggtaaacagcagaaa-3' (reverse primer); D6S2222 5'-agtcatctgaagagttgg-3' (forward primer) and 5'-gcatgtcttctttgttaagg-3' (reverse primer); for D6S105 5'-gggattacaggcaggagccac-3' (forward primer) and 5'-gaaggagaattgtaattccg-3' (reverse primer) and for D6S2239 5'-gttggaagcaatggattagatgtcc-3' (forward primer) and 5'-ctacctgccaggaacaatatacac-3' (reverse primer). Amplification was performed according to an established protocol. Briefly, Ampli*taq *gold (Perkin-Elmer) was used to amplify specific amplicons, using genomic DNA as template. Amplification was performed, in all cases, with an initial denaturation step of 94°C (8 minutes), followed by 30 cycles at 94°C (15 sec.), 55°C (15 sec.) and 72°C (30 sec.), and a final extension step of 30 minutes at 72°C. Alleles were identified by electrophoresis on sequencing gels. The classification adopted for each microsatellite allele is based on the size (in base pairs) of each DNA fragment amplified.

### Statistical considerations

Group means were compared by the Student T-test when two groups were analyzed or by the one-way analysis of variance test (ANOVA) when more than two groups were analyzed. The Chi-square test was used to test the fitness of data to the normal distribution. Independence between categorical data and differences between allele frequencies of the genetic markers between the hemochromatosis patients and the control population were tested using the Chi-Square test.

Two different sets of analyses were done in C282Y homozygous subjects to investigate the impact of the genetic markers from the 6p21.3 region, on the CD8+ T-lymphocyte numbers. First, a single-locus analysis was performed by one-way ANOVA, where the numbers of CD8+ T lymphocytes were analyzed in relation to the alleles of each genetic marker studied (HLA-A, -B and -C and microsatellites D6S265, D6S2222, D6S105 and D6S2239). Single-locus analyses were also done for CD4+ T-lymphocyte and total lymphocyte numbers. Since each subject carries two chromosomes and therefore two different allele specificities, a second set of analysis was done considering the combination of the two inherited HLA-A alleles. For this purpose a trichotomous variable denominated "HLA-A genotype" (details in the result' section), was created and its impact on the variability of CD8+ T lymphocytes was also analyzed using a stepwise multiple regression analysis. In this model, CD8+ T lymphocytes were set as dependent variable and the HLA-A genotype, age, gender and iron measures (TfSat, serum ferritin and TBIS) were used as independent variables. Because serum ferritin values were not normally distributed, a log transformation was used for this variable.

To investigate the effect of the "HLA-A genotype" on the severity of the disease, in terms of iron stores (TBIS), a stepwise multiple regression model was used. TBIS was set as dependent variable and the HLA-A genotype as independent variable, together with age and gender. The interaction of the variable gender with the other independent variables was tested by introducing two dummy variables in the model, corresponding to male or female gender.

The overall fit of ANOVA is indicated by *F *and *p *values. The overall fit of multiple regression model is indicated by the full regression *R*^2^, and the *F *and *p *values of each variable in the model. All statistical tests were performed at the 0.05 level of significance and all *p *values are two-sided. Data were analyzed by Statgraphics software (Statgraphics Graphics System, version 7.0).

## Results

### General characterization of C282Y homozygous probands

In the group of 43 C282Y homozygous probands all had TfSat above 65% at the time of the diagnosis. There was, however, a great variability in serum ferritin levels and also in the amount of iron removed by phlebotomies (TBIS) (Table 1). Although males presented with higher average values for TfSat, serum ferritin and TBIS than females, values only reached statistical significance for TBIS (Table 1). No significant differences were observed in the numbers of total lymphocytes and CD4+ and CD8+ T lymphocytes between males and females, although the lowest values of CD8+ T-cell counts were observed in males (Table 1). The proportion of patients who showed CD8+ counts below the median (previously established in a control population = 0.41 × 10^6^/ml [12]), was 75% (24/32) in males and 36% (4/11) in females.

### Genetic characterization of C282Y homozygous probands

#### HLA A-B haplotypes

**Figure 2:**
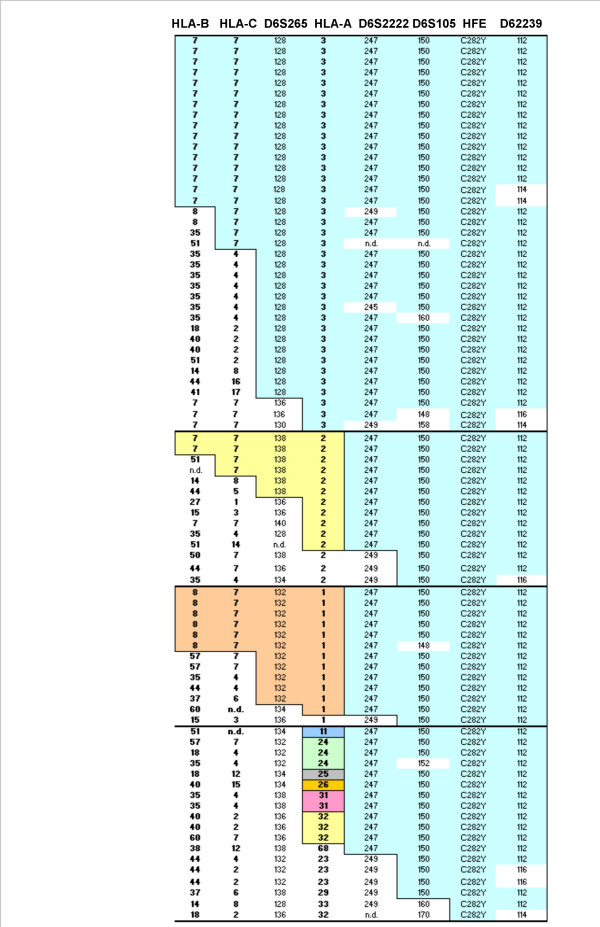
Multi-locus map of C282Y carrying haplotypes from a region spanning 5 Megabases from HLA-B to D6S2239. The allele specificity in each locus is represented by numbers corresponding to the size of the amplified DNA fragments. Color boxes represent conserved haplotype areas. n.d.: not determined.

A total of 86 HLA A-B haplotypes were defined by segregation in families from the 43 homozygous probands included in the study. Thirty-seven different HLA A-B haplotypes could be identified. These are shown in Figure 2. As expected, the haplotype A*03-B*07 was the most common (22/86; 0.256), being present in 44% (19/43) of the subjects, 16 in heterozygozity and 3 in homozygozity. The next most common haplotypes were the A*03-B*35 (7/86; 0.081) and A*01-B*08 (6/86; 0.070), followed by A*02-B*51 and A*02-B*07 (each haplotype present in 4/86; 0.047). Haplotypes A*02-B*44, A*23-B*44 and A*32-B*60 were present in three probands each (3/86; 0.035). All the other 28 haplotypes had frequencies below 0.025 and 22 of the haplotypes were represented only once (see Figure 2).

#### Extended HLA haplotypes

A total of 82 extended HLA haplotypes could be defined from the 43 hemochromatosis probands (see Methods). Haplotypes were aligned taking into account the HLA-A and then arbitrarily according to the similarity of the alleles from the several microsatellites. The predominant extended HLA haplotype carries the HLA-A*03-B*07 and is defined by: HLA-B*07, HLA-C*07, D6S265-128, HLA-A*03, D6S2222-247, D6S105-150, *HFE*-C282Y, D6S2239-112 (Figure 2). This haplotype is present in 14/82 (17%) of subjects. Clearly, the most conserved region spans from HLA-A to D6S2239, where the A*03 carrying haplotypes defined by HLA-A*03, D6S2222-247, D6S105-150, *HFE*-C282Y, D6S2239-112 is present in 35% (29/82) of the chromosomes (Figure 2). Two other common haplotypes, carrying the HLA-A*02 and A*01, were found. The haplotype HLA-A*02, D6S2222-247, D6S105-150, *HFE*-C282Y, D6S2239-112 is present in 13% (11/82) and the haplotype HLA-A*01, D6S2222-247, D6S105-150, *HFE*-C282Y, D6S2239-112 is also present in 13% (11/82) of the chromosomes (Figure 2). The higher frequency of the HLA-A*03, -A*02 and -A*01 carrying haplotypes, as well as their relative lower microsatellite diversity (illustrated in Figure 2) suggest that these haplotypes are older in the evolutionary history of the C282Y mutation in this population.

### Single-locus analysis of genetic markers from the MHC region in relation to CD8+ T lymphocytes from C282Y homozygous probands

**Figure 3:**
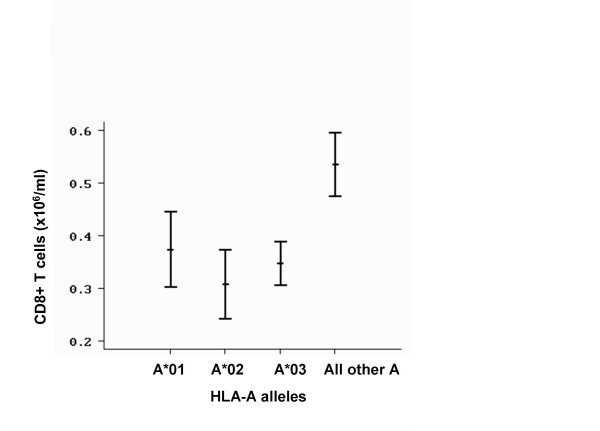
Mean values ± standard error of the mean of the CD8+ T-lymphocyte numbers according to the presence of different HLA-A alleles among haplotypes carrying the C282Y mutation. Haplotypes carrying the HLA-A*01 are 13, the A*02 are 16 and the A*03 are 39. The group of "All other A" specificities include: HLA-A*11, A*23, A*24, A*25, A*26, A*29, A*31, A*32, A*33 and A*68 and are 18.

The numbers of CD8+ T lymphocytes were analyzed in relation to single-locus HLA-A, -B, -C alleles and microsatellites D6S265, D6S2222, D6S105, D6S2239 alleles, by one-way ANOVA. A statistically significant effect (*F *= 2.36, *p *= 0.013, one-way ANOVA) of the HLA-A alleles on the CD8+ T-cell numbers was found. Among all HLA-A specificities, the A*03 (n = 39), A*02 (n = 16) and A*01 (n = 13) were associated with the lowest average CD8+ T-cell counts. This effect is more clearly evidenced when all chromosomes carrying HLA-A alleles other than A*03, A*02 and A*01 (chromosomes carrying A*11, A*23, A*24, A*25, A*26, A*29, A*31, A*32, A*33 and A*68; n = 18) are pooled together in one group as illustrated in Figure 3 (*F *= 5.58, *p *= 0.0016, one-way ANOVA). No statistically significant associations were found with any of the more centromeric markers, D6S265, HLA-C and HLA-B. No significant associations were also found with the telomeric markers D6S2222, D6S105 and D6S2239, an expected result taking into consideration the high conservation of the region as evidenced by the presence of the same microsatellite alleles in the large majority of C282Y carrying chromosomes.

A statistically significant result (*F *= 3.21, *p *= 0.027, one-way ANOVA) was observed when the effect of the HLA-A was tested on total lymphocyte counts (data not shown). No statistically significant associations were found for total lymphocyte counts with any of the other markers used. No statistically significant effect was found on the numbers of CD4+ T cells and any of the genetic markers tested (data not shown).

### The combined effect of the two inherited HLA-A alleles on the CD8+ T-lymphocyte numbers from C282Y homozygous probands

To further investigate the association found between particular HLA-A alleles in chromosomes carrying the C282Y mutation and the number of CD8+ T cells, C282Y homozygous subjects were divided according to the combination of the two inherited HLA-A alleles. This created a trichotomous variable, from now on denominated "HLA-A genotype", dividing C282Y homozygous probands in the following groups: 1.subjects with two copies of the most common HLA-A alleles (A*03, A*02 and A*01; n = 27); 2.subjects with only one copy of the most common HLA-A alleles (n = 14) and 3.subjects without any copy of those alleles (n = 2). A stepwise multiple regression analysis was run, where the number of CD8+ T lymphocytes was set as dependent variable and the HLA-A genotype, iron measures (TBIS, TfSat and serum ferritin), age and gender (dummy variable), were set as independent variables. The only variable significantly affecting CD8+ T-cell numbers was the HLA-A genotype (*F *= 20.56 and *p *= 0.0001). The *R*^*2 *^for the full regression was 0.37. Iron related variables did not enter the model (*F *= 1.78 for TBIS; *F *= 0. 848 for TfSat and *F *= 0.201 for ferritin). Gender and age also did not enter the model (*F *= 1.16 and *F *= 0.123, respectively). Due to the fact that there are only two subjects without any copy of the most common HLA-A alleles and these two had the highest CD8+ numbers, a new regression was run where the HLA-A genotype variable was analyzed only with the groups of subjects with either two copies of the most common HLA-A alleles (n = 27) or with only one copy of those alleles (n = 14). A statistically significant effect of the HLA-A genotype on CD8+ T-cell numbers was maintained (*F *= 7.98 and *p *= 0.0082).

**Figure 4:**
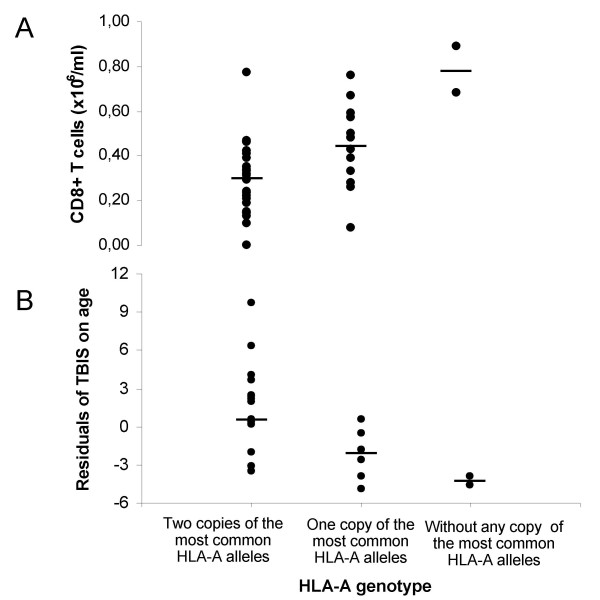
Distribution of the CD8+ T-lymphocyte numbers (A) and of the total body iron store (TBIS) after correction for the effect of age (residuals of TBIS on age) (B), in males, according to the HLA-A genotype of the C282Y homozygous probands, which divide subjects in the three groups: 1.subjects with two copies of the most common HLA-A alleles (A*03, A*02 and A*01; n = 27); 2.subjects with only one copy of the most common HLA-A alleles (n = 14) and 3.subjects without any copy of those alleles (n = 2).

The effect of the HLA-A genotype on CD8+ T lymphocytes is illustrated in Figure 4A. Subjects carrying two copies of the most common HLA-A alleles (A*03, A*02, A*01) presented the lowest average CD8+ T-cell numbers (0.30 ± 0.14 × 10^6^/ml) and subjects without any copy of the most common HLA-A alleles presented the highest average value (0.79 ± 0.15 × 10^6^/ml). Subjects with only one copy of the most common HLA-A alleles presented intermediate average CD8+ T-cell numbers (0.46 ± 0.19 × 10^6^/ml).

### The effect of HLA-A alleles on the CD8+ T-lymphocyte numbers from control subjects

The results described in C282Y homozygous probands confirm an association of the HLA-A region with the setting of CD8+ T lymphocytes. Since CD8+ T lymphocytes are known to expand in the context of MHC class I molecules, it is plausible to consider that the HLA specificity itself could be responsible for the results observed. To test this hypothesis, we examined retrospectively the effect of the combinations of HLA-A alleles on CD8+ T-cell numbers in a previously studied control population, not carrying the C282Y mutation [12]. Control subjects were grouped according to the same combinations of alleles used in C282Y homozygous subjects. In contrast with the C282Y homozygous population, no differences were observed among control subjects carrying combinations of A1, A2 or A3 alleles (0.47 ± 0.14 × 10^6^/ml, n = 65), control subjects carrying only one copy of these alleles (0.45 ± 0.21 × 10^6^/ml, n = 31) and subjects without any copy of those alleles (0.41 ± 0.17 × 10^6^/ml, n = 14), therefore not supporting a direct effect of the HLA specificity on the results. In the previously described control population [12], subjects carrying the HLA-A*01 had significantly higher CD8+ T-lymphocyte numbers, a result apparently in contradiction with what was described in the present work, where C282Y homozygous probands carrying the A*01 have lower CD8+ T-cell counts. To address this question we genotyped control subjects carrying the HLA-A*01 (n = 47) for the microsatellites D6S2222 and D6S105 and calculated frequencies of the alleles 247 and 150, which were found consistently associated in the haplotypes carrying the C282Y mutation (allele frequencies are respectively 0.875 and 0.958). In contrast with C282Y homozygous, the most commonly found allele of the microsatellite D6S2222 in control subjects carrying the HLA-A*01, was not the 247 (32/94; allele frequency = 0.340) but rather the 249 (45/94; allele frequency = 0.479). This difference in allele frequencies was statistically significant (*p *< 0.00001). For the microsatellite D6S105, although the allele 150 was the most common in both groups, its allele frequency was significantly lower (*p *= 0.0001) in controls (47/94; allele frequency = 0.500) than in C282Y homozygous (23/24; allele frequency = 0.958).

### Impact of the HLA-A genotype on the clinical expression of HH

In face of the results showing an impact of the HLA-A region on the setting of the CD8+ T-cell numbers and the previously described association of low CD8+ T cells with a more severe expression of iron overload [16,17], we reinforce the hypothesis that a putative gene controlling CD8+ T-lymphocyte numbers could be a modifier of the clinical expression in hemochromatosis [12]. To further investigate this hypothesis we tested the effect of the HLA-A genotype in the levels of TBIS by stepwise multiple regression analysis. For this purpose, the variable TBIS was taken as dependent and HLA-A genotype, age and gender (dummy variables) as independent variables. The variables entering into the model were age and HLA-A genotype only in males (*F *= 17.29 and *F *= 7.16, respectively). The *R*^*2 *^for the full regression was 0.306 and the global significance level was 0.0009. This result is illustrated by the correlation between HLA-A genotype and residual values of TBIS on age in males, as shown in Figure 4B. However, if the number of CD8+ T lymphocytes is introduced in the model, the effect of HLA-A genotype is lost, and the only significant variables entering the model are age and CD8+ T cells in males (*F *= 16.54 and *F *= 7.58, respectively). The *R*^2 ^for the full regression is similar (0.303; *p *= 0.0012). This result shows that the effect of the HLA-A region on the severity of iron stores may be confounded by the number of CD8+ T lymphocytes.

## Discussion

The present study was designed to further investigate our previous results showing that CD8+ T-lymphocyte numbers are under the control of genes localized at the MHC region. An attempt to narrow the candidate region is made, using as a model 43 HH subjects, C282Y homozygous, in whom a high proportion of subjects have low CD8 + T-cell numbers. The results provide evidence supporting an inextricable link between extended HLA haplotypes, CD8+ T-lymphocyte numbers and severity of iron overload in HH.

### The HLA-A region is associated with the low CD8+ T-lymphocyte trait in HH

Single-locus analysis of the several genetic markers of the MHC region in chromosomes carrying the C282Y mutation showed that CD8+ T-lymphocyte numbers were associated with the HLA-A region. No significant association was found with the more telomeric markers, spanning from the microsatellites D6S2222 to D6S2239. This region is well conserved in all haplotypes, independently of CD8+ T-cell numbers. Centromerically to HLA-A, a high allele diversity is observed and no association was found with CD8+ T-cell numbers (Figure 2). Therefore, a putative genetic marker of CD8+ T-lymphocyte numbers could be localized between D6S2222 and HLA-A. Since each individual, with a particular CD8+ T-cell profile, has two different HLA-A alleles, it may be expected that the CD8+ profile will be the result of the combined effect of the two chromosomes. Indeed, we found that, in the C282Y background, the inheritance of two of the most common HLA-A alleles (A*03, A*02 and A*01) is associated with a low CD8+ T-lymphocyte profile and the inheritance of only one of those alleles is associated with higher CD8+ T-lymphocyte profile (0.30 ± 0.14 versus 0.46 ± 0.19 × 10^6^/ml). The highest value of CD8+ T-cell numbers was observed in the subjects without any copy of the most common HLA-A alleles (0.79 ± 0.15 × 10^6^/ml). The most attractive hypothesis to explain these results is the existence of a genetic trait associated with low CD8+ T-lymphocyte numbers localized in the HLA-A region. In this case, the trait would be in linkage with haplotypes carrying the A*03, A*02 or A*01 in the C282Y background. In a previous report, Pratiwi and collaborators [24] showed, by an extended linkage disequilibrium analysis in the hemochromatosis gene region, two distinct peaks of association separated by 2 Megabases and referred that "such did not describe the expected pattern for a single gene disorder" [24]. The authors found a highly significant association at D6S2239, localized in close proximity to *HFE *(14 kilobases telomeric) and at D6S105, located about 200 kilobases away from the D6S2222 [24]. The possibility that the results described by Pratiwi and co-workers correspond to the presence of the same genetic trait described here is highly attractive. The finding of low CD8+ T-cell counts associated with HLA-A*01 carrying haplotypes is also in accordance with the recently reported association of low total lymphocyte counts with the HLA haplotype A*01-B*08 in an independent population of hemochromatosis patients from Alabama [30].

The possibility that the present results are due to the effect of the HLA specificity itself must be considered. In a control population not carrying the C282Y mutation, however, combinations of the same HLA-A alleles, as observed in patients, did not reveal the same association with CD8+ T-cell numbers, constituting an argument against this possibility. This does not exclude a possible interaction between HFE and HLA, as recently suggested in a study of MHC class I expression in peripheral blood mononuclear cells from patients carrying the C282Y mutation [31].

The association of low CD8+ T-lymphocyte counts with HLA-A*01 in C282Y carrying subjects, seemed to be in contradiction with our own previous work, where it was shown that the majority of HLA-A*01 normal subjects had high numbers of CD8+ T lymphocytes [12]. However, it is likely that the association with high or low CD8+ T-cell counts is not dependent on the HLA-A*01 specificity itself but rather on another putative marker inherited in linkage with particular haplotypes, depending on the recombination history of the region. Microsatellite markers in the region close to HLA-A (D6S2222 and D6S105) showed that the HLA-A*01 carrying haplotypes are not the same in chromosomes carrying or not the C282Y mutation. In the case of A*01, D6S2222-247, D6S105-150, C282Y haplotypes, there is a clear association with low CD8+ T-lymphocyte counts, in contrast with A*01 haplotypes in the normal population that do not carry the C282Y mutation. These results support the hypothesis that inherited mutations in a putative gene controlling CD8+ T-cell numbers could be differentially transmitted in chromosomes carrying or not the C282Y mutation.

### Implications on the clinical heterogeneity of HH

The inheritance of different HLA-A alleles also influenced the clinical severity of the disease, in terms of iron removed by phlebotomy. Multivariate analysis of TBIS with HLA-A genotype, age and gender, showed an association of the HLA-A genotype with TBIS and in particular with the degree of iron accumulation with age in males (Figure 4B), in accordance with results previously reported by other authors [32-35]. In addition, in a multiple regression model it was shown a stronger effect of the CD8+ T-lymphocyte counts in accordance with our own previously reported data [16]. Altogether the results support the notion that gene(s) in this region contribute both to the control of CD8+ T-cell numbers and the clinical heterogeneity observed in HH (Figure 4A and 4B). The question remains if these associations are explained by one single gene or by independent genes in linkage disequilibrium in the same locus. The search for possible recombinants defined by genotype/phenotype discrepancies may help in future to elucidate this question.

### Evolutionary hypothesis concerning a genetic trait of low CD8+ T lymphocytes in HH subjects

Based on the definition of 82 extended HLA haplotypes in C282Y homozygous subjects we would like to advance an evolutionary hypothesis to explain the high frequency of abnormally low CD8+ T-lymphocyte numbers in HH. The predominant extended HLA haplotype found in the present population, carries the HLA A*03-B*07 (Figure 2), assumed to be the ancestral haplotype where the mutation was originated. We found a clear association between low CD8+ T-cell counts and the HLA-A*03, suggesting that the original chromosome where the C282Y appeared carried the A*03-B*07 and also the genetic trait associated with low CD8+ T lymphocytes. As described before by Simon and collaborators [36] recombination events of the HH haplotypes, first occurred centromerically to HLA-A, originating a multiplicity of HLA-A*03 carrying haplotypes. Recombination between the ancestral haplotype and rare haplotypes in the population are less likely to occur than recombinations between an ancestral haplotype and common haplotypes. Thus, it is likely that more telomeric recombinations introduced the HLA-A*02 and -A*01 alleles, the most common alleles found in the control population [12], giving rise to A*02 and A*01 haplotypes carrying the C282Y mutation that, conceivably, are also ancestral haplotypes in the evolutionary history of the C282Y mutation. These haplotypes still maintain the characteristic of low CD8+ T lymphocytes considering that the putative gene controlling these numbers is localized telomerically to HLA-A. Subsequent recombination events between HLA-A and the D6S2222 may lead to the appearance in the C282Y carrying haplotypes of HLA-A alleles less frequently found in the control population (A*11, A*23, A*24, A*25, A*26, A*29, A*31, A*32, A*33, A*68), and to the disruption of the linkage with the putative genetic trait of low CD8+ T lymphocytes. It is possible that haplotypes carrying these HLA-A alleles are more recent in the evolutionary history of the C282Y mutation. In the light of the present evolutionary hypothesis it is intriguing why chromosomes bearing *HFE*-C282Y and HLA-A*01, -A*02 or -A*03 alleles may have become relatively common in the general population, considering that low numbers of CD8+ T lymphocytes do not seem to be an advantageous trait. One could speculate that a selective advantage of these haplotypes should be only observed in heterozygozity, i.e., in combination with other diverse haplotypes. In this context it would also be interesting to know which haplotypes are found in the vast number of apparently non-expressing C282Y homozygous recently found in large population screenings [37,38].

### Implications of the evolutionary hypothesis on the clinical expression of HH

The evolutionary hypothesis proposed above could explain the association found by others between the HLA region and the iron overload. Studies conducted before and after the discovery of the *HFE*, have shown a more severe phenotype in patients carrying the ancestral HLA haplotype A*03-B*07 or the HLA allele A*03 [16,19,32-35]. Two independent studies where the number of copies of the ancestral haplotype was analyzed showed a more severe expression in patients carrying two copies of the ancestral haplotype as compared to those with one copy and those without [19,33].

The hypothesis advanced here does not explain differences between males and females. It is well known that males are predominantly affected and present a more severe iron accumulation [16,22,35], as also shown here, and that the correlation of CD8+ T cells with iron stores is only observed in males [16]. An attempt to explain gender differences was recently reported based on different frequencies of the HLA alleles and haplotypes [39]. Although significant differences in the haplotype frequencies of A*03-B*07 between males and females were found, these did not correspond to differences in iron measures [39]. It is possible that whatever the mechanisms involved in the relationship between HLA, CD8+ T cells and iron overload, it is not so relevant in females as in males because other factors may be involved. The recent description that serum transferrin is a limiting resource for the size of the MHC class I dependent lymphocyte pool, and that the lowest transferrin levels are observed in males, may help to explain the increased susceptibility of males to iron overload [40].

## Conclusion

There are now intensive efforts to understand the genetic mechanisms involved in the control of T-lymphocyte numbers [10,11]. The present results constitute a step forward to narrowing the region in chromosome 6 to look for a candidate gene involved in the regulation of CD8+ T-lymphocyte numbers. Further studies with extended genetic markers in the region may in future elucidate this question. In addition, the present study corroborates the previous hypothesis that gene(s) contributing to the clinical heterogeneity observed in HH could be genes that also control CD8+ T-cell numbers. The identification of a putative gene controlling the CD8+ T-lymphocyte numbers may be of great relevance not only in the management of hemochromatosis but also in other clinical situations where iron and lymphocytes may be important pathologic co-factors, such as in cancer and infection.

## Competing interests

The author(s) declare that they have no competing interests.

## Authors' contributions

EC and GP conceived and designed the study, diagnosed and treated the hemochromatosis patients, compiled their clinical data, contribute to the interpretation of data and wrote the manuscript. Additionally, EC analyzed the data and performed the statistical analysis related to hemochromatosis patients and family members; compiled data and performed statistical analysis related to the control population. JV participated in the design of the study and contributed to the statistical analysis and its interpretation and to the writing of the manuscript. SA performed the *HFE *genotyping. RL performed the T-cell immunophenotyping. AG and CSC performed the microsatellite genotyping and some of the HLA genotyping. HA is responsible for the HLA genotyping and contributed to the interpretation of the genetic data. All authors read and approved the final manuscript.

## Pre-publication history

The pre-publication history for this paper can be accessed here:


